# Electronic Cigarette or Vaping-Associated Lung Injury Case Report

**DOI:** 10.21980/J8S65P

**Published:** 2023-01-31

**Authors:** Amy Chuang, Lauren Bacon, Anthony Lucero

**Affiliations:** * Kaweah Healthcare District, Department of Emergency Medicine, Visalia, CA

## Abstract

Electronic cigarette (e-cigarette) or vaping associated lung injury (EVALI) cases have increased with the popularity of e-cigarettes in the mostly young, healthy population. Some common symptoms associated with EVALI include shortness of breath and chest pain, and the most common diagnostic imaging findings are organizing pneumonia and diffuse alveolar damage seen on computed tomography (CT). Pneumomediastinum is a known sequela of EVALI.^1^ In the setting of pneumomediastinum in EVALI, EVALI is a diagnosis of exclusion, so other sources of pneumomediastinum need to be evaluated. EVALI has diverse presentations, and this case is a unique representation of a disease process that is becoming more commonplace with the increase in popularity of vaping. It is important to be aware of the clinical symptoms of EVALI, which can be nonspecific and can include gastrointestinal symptoms along with respiratory symptoms. It is equally important to recognize the diverse image findings of EVALI, which can include subcutaneous emphysema and pneumomediastinum. In this case, pneumomediastinum is seen in EVALI, and the patient was successfully treated with empiric antibiotic coverage, steroids, and conservative measures— making sure to limit any coughing or increases in intrathoracic pressure that can cause worsening of pneumomediastinum.

**Topics:**

EVALI, vaping, pneumomediastinum, E-cigarette, ground-glass opacity.

**Figure f1-jetem-8-1-v22:** Video Link: https://youtu.be/FPEDuXs0TFo

## Brief introduction

[Fig f1-jetem-8-1-v22]E-cigarettes and vaping devices have become more popular among young populations due to perceived low risk of harm. Each device consists of a battery, a cartridge containing liquid, and a coil, which vaporizes the liquid, which can then be inhaled.^2^ Clinical presentation can vary, but usually consists of a constellation of respiratory, gastrointestinal, and systemic symptoms.^3^ The diagnosis of EVALI is one of exclusion and depends on various factors including: 1) e-cigarette/vaping use within the last 90 days, 2) abnormal chest imaging, 3) ruling out infections, cancer, chemical exposures, and autoimmune diseases.^4^ Imaging findings can range from organized pneumonia, diffuse alveolar damage, diffuse alveolar hemorrhage, mild nonspecific inflammation, to granulomatous pneumonitis, etc.^4^ In this case, we present a 25-year-old male with recent vaping who developed diffuse pneumomediastinum and pneumopericardium as a rare complication of EVALI.

## Presenting concerns and clinical findings

25-year-old male with a past medical history of Bipolar, Type 1 presented to the ED in the summer of 2021 for evaluation of difficulty breathing for two weeks. He had associated chest discomfort with shortness of breath, reporting that he felt “Rice Krispies in my chest.” Due to discomfort, he had been unable to sleep. He admitted to smoking marijuana but denied smoking cigarettes. He also admitted to recently starting vaping about 4 weeks ago, using roughly 3 tetrahydrocannabinol cartridges during this time. He denied sick contacts at home. Patient stated that he had a “summer cold” roughly 3–4 weeks ago. He denied fever, chills and rhinorrhea; however, he did admit to coughing.

Initial vital signs showed core temperature of 36.6 degrees Celsius, blood pressure of 127/83, heart rate of 114, respiratory rate of 25 and oxygen saturation of 96% on room air. On the physical exam, he was cooperative, but displayed a disorganized thought process with rapid speech. His airway was patent, and there was no increased work of breathing. His lungs were clear to auscultation bilaterally with good air movement. His heart was tachycardic, with no murmurs. Diffuse crepitus was palpated on the chest, abdomen, and neck.

In the ED, the patient was positive for opiate and cannabinoid use on urine drug screen. Swabs for influenza and coronavirus were negative. Chest radiograph (X-ray) showed diffuse subcutaneous emphysema, with diffuse lung opacity present and possible apical pneumothorax. Vancomycin and piperacillin-tazobactam were given in ED for empiric coverage for bacterial pneumonia. Given the chest X-ray findings as well as patient’s shortness of breath, a CT of chest with contrast was ordered to evaluate for etiology of subcutaneous emphysema. The patient was admitted to step-down level of care for pneumomediastinum and interstitial pneumonia.

## Significant findings

The CT of the chest with contrast showed subcutaneous emphysema (green star), pneumomediastinum (yellow arrow), and pneumopericardium (purple asterix) without an identifiable tracheal tear. Extensive air was visualized as hypodense areas within the chest wall within the soft tissue. The image also detailed a hypodense area surrounding the heart consistent with pneumopericardium. No disruption of the trachea was present. Additionally, the CT of the chest also showed bilateral ground glass airspace opacities (red stars) with subpleural sparing that is consistent with EVALI findings.^2^,^5^ These specific findings have been seen in many of the EVALI cases.^5^ This image is interesting because there is extensive pneumomediastinum with no clearly identifiable cause. The imaging shows no esophageal or tracheal or lung injury, so it is important to note relevant information collected during interview regarding patient’s recent history of vaping THC, especially when establishing a differential diagnosis.

## Patient course

Upon further assessment, the patient’s mother mentioned that the patient left an outside hospital about two days ago, and the patient told her he had “air leaking out,” a statement the patient’s mother was unable to clarify the meaning of. Chart review of medical records from the outside hospital showed that he was admitted for acute respiratory failure with hypoxia, and left against medical advice two days prior to admission at the final facility. At the outside facility, the patient was found to have a large pneumomediastinum. Chest X-ray showed “diffuse consolidation and apicobasal gradient and air bronchogram.” His d-dimer was elevated, but subsequent CT with angiogram of the chest was negative for a pulmonary embolism; however, it did show “multifocal ground-glass airspace opacities with prominent subpleural sparing.” In the outside facility, his rapid coronavirus test was negative, and the patient was admitted to hospital and started on azithromycin and rocephin for empiric treatment of pneumonia. Initial vitals at the outside facility showed that the patient was hypoxic to 87% and was subsequently placed on oxygen at 3 liters nasal cannula. Patient had reported that he started vaping about 1 month ago when his symptoms began, and used 3 tetrahydrocannabinol cartridges during that time.

During the patient’s previous admission at the outside facility, a radiograph esophagram was performed which showed no evidence of esophageal leak, no gastric reflux, and only a small hiatal hernia was visualized. Two sets of blood cultures performed there showed no growth after 2 days. Patient was also negative for Strep Pneumoniae, legionella, and mycoplasma, with a negative viral respiratory panel. Pulmonology evaluated the patient and recommended prednisone 60 mg twice a day for 5 days, then taper. Cardiothoracic surgery recommended against any surgical intervention. The patient had elevated C-reactive protein (CRP) at 389 mg/dL, lactate dehydrogenase (LDH) at 94 Units/L, ferritin at 816.5 ng/mL, D-dimer at 2657 ng/mL, procalcitonin at 0.32 ng/mL. He had a leukocytosis of 18.9 x 10^3^/mm^3^. Patient was also hypokalemic at 3.2 mEq/L, which was repleted accordingly. Tessalon pearls were given for cough suppression.

Two days after leaving the outside hospital against medical advice, he presented to our ED and was admitted at our facility. During the patient’s admission, he was continued on rocephin and azithromycin for empiric coverage of possible community acquired pneumonia. Prednisone was continued 60 mg for 5 days tapered to 10 mg a day for 6 weeks to prevent coughing and worsening of pneumomediastinum. Bilateral ultrasound doppler of lower extremities showed no evidence of deep venous thrombosis. Repeat blood cultures showed no growth after 5 days. The patient was given ipratropium/albuterol, with antitussive medications as needed, to prevent increased intrathoracic pressure via coughing. Repeat chest X-ray shows “extensive subcutaneous emphysema along bilateral chest walls” and “airspace opacities in bibasilar region.” The patient was downgraded to Medical/Surgical floor after the first day. Psychiatry team was also consulted due to the patient’s odd behaviors and tangential speech, who recommended quetiapine for treatment of underlying Bipolar disorder.

The patient was initially placed on 2 liters oxygen via nasal cannula for comfort, but was eventually weaned off. During the patient’s stay, existing leukocytosis trended up, consistent with prednisone use. Blood work also showed thrombocytosis which is most likely reactive. Lactate trended down from 0.8 to 0.17 mmol/L. The patient was switched to oral cefuroxime. Psychiatry was consulted and stated that the patient did not meet criteria for 5150 hold, and the patient was discharged under care of his mother for close follow up with outpatient psychiatry. He was discharged with a prescription for quetiapine and prednisone taper.

## Discussion

The Center for Disease Control formulated a diagnosis of EVALI as: 1) Vaping history within 90 days before symptom onset, 2) Pulmonary infiltrate or ground glass opacities on chest X-ray or chest CT, 3) Negative for pulmonary infection, with minimum of negative respiratory viral panel and influenza PCR, 4) No evidence of an autoimmune disease or malignant process.^2^ Given these definitions, the patient met criteria for EVALI diagnosis. However, ground glass opacities with subpleural sparing is a nonspecific finding that can be seen in other lung pathologies including COVID, pulmonary edema, pneumocystis jirovecii pneumonia, pulmonary contusion, etc.^6^

In this unique case, the patient had a pneumomediastinum with ground glass opacities. Although there have been some rare case studies showing a similar pattern of vaping induced pneumomediastinum, CT of chest in this case showed extensive pneumomediastinum as well as pneumopericardium.^7^,^8^,^9^,^10^ This image highlights many findings that can be seen in EVALI, including ground glass opacities with subpleural sparing as well as pneumomediastinum and pneumopericardium. Pneumomediastinum secondary to EVALI needs to be a diagnosis of exclusion in the setting of no other causes of

pneumomediastinum, including pneumothorax, esophageal rupture, or tracheobronchial tree rupture. For this case, X-ray esophagram was performed to assess for possible esophageal perforation, which was negative. CT of chest showed no identifiable tracheal tear; however, no bronchoscopy was performed on this patient at either facility. When reviewing the notes, it is unclear why no bronchoscopy was performed, and this is a limitation because we are unable to definitively rule out any tracheobronchial tears or ruptures that could have caused the pneumomediastinum. Another limitation to this case report is that no bronchoalveolar lavage (BAL) was done, and even if one was done, obtaining a Vitamin E acetate level would not have been possible since neither our hospital laboratory, nor the laboratory it is contracted with for send-out labs, have the assay for that test. There have been studies that have shown an association in cases of EVALI and BALs with increased Vitamin E acetate, and Vitamin E acetate has been postulated to be the cartridge additive affecting pulmonary surfactant and causing EVALI.^11^ A strength of the case is that the patient was thoroughly worked up for infectious etiology, including viral respiratory panel, COVID/Flu testing, and sputum cultures, all which were unremarkable.

Spontaneous pneumomediastinum can be caused by an increase in intrathoracic pressure, including coughing or bearing down. Increase in intrathoracic pressure from coughing or inhaled drug use can cause alveolar rupture, leading to dissection of air through bronchovascular sheaths.^9^ When vaping, over inhaling often occurs, requiring forceful exhalation and an increase in intrathoracic pressure, leading to possible spontaneous pneumomediastinum.^8^ Common patterns of lung injury seen on imaging include organizing pneumonia, diffuse alveolar damage, acute eosinophilic pneumonia, and diffuse alveolar hemorrhage.^4^ If lung injury is noted on imaging, as in this case, ground glass opacities seen with subpleural sparing, steroids have been proven to be effective in preventing further progression of lung injury.^4^ Once pneumomediastinum secondary to EVALI is diagnosed, patients need to be admitted for monitoring and treatment including avoiding triggers and providing oxygen, pain control, and bed rest.^9^ The main learning points of the case is how to recognize EVALI, what the minimum necessary diagnostic and imaging studies are to diagnose EVALI, and the necessity of ruling out pneumomediastinum secondary to other causes first.

## Supplementary Information








