# Supplementary figures and images for: Electronic Cigarette or Vaping-Associated Lung Injury Case Report
Source: J Educ Teach Emerg Med. 2023 Jan 31;8(1):V22–7. doi: 10.21980/J8S65P (PMC10332769; doi:10.21980/J8S65P)

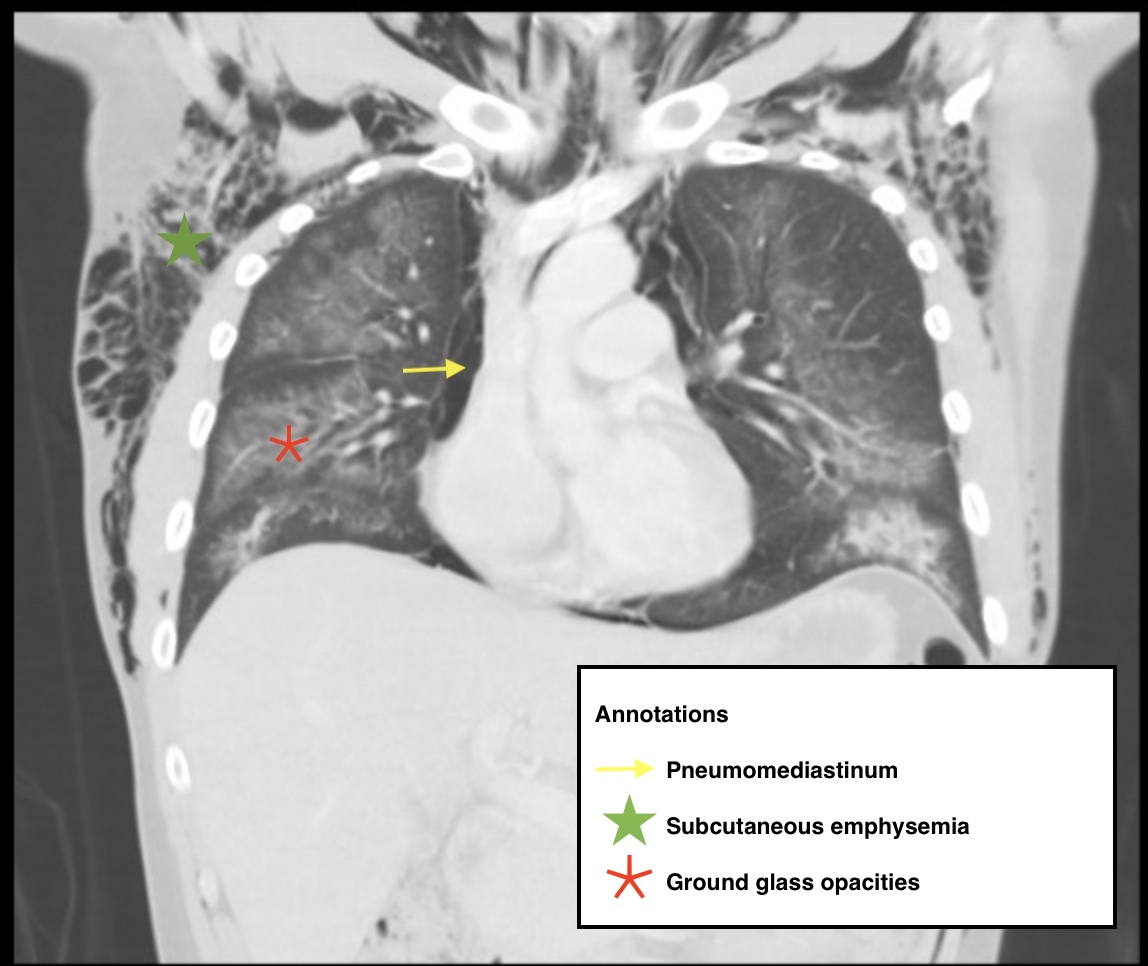

Supplement: Supplementary file 2 [file jetem-8-1-v22-supp1.jpg]

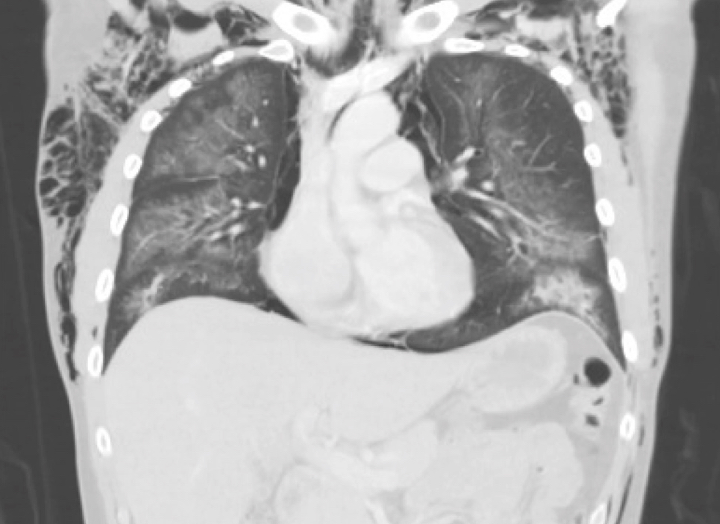

Supplement: Supplementary file 3 [file jetem-8-1-v22-supp2.jpg]

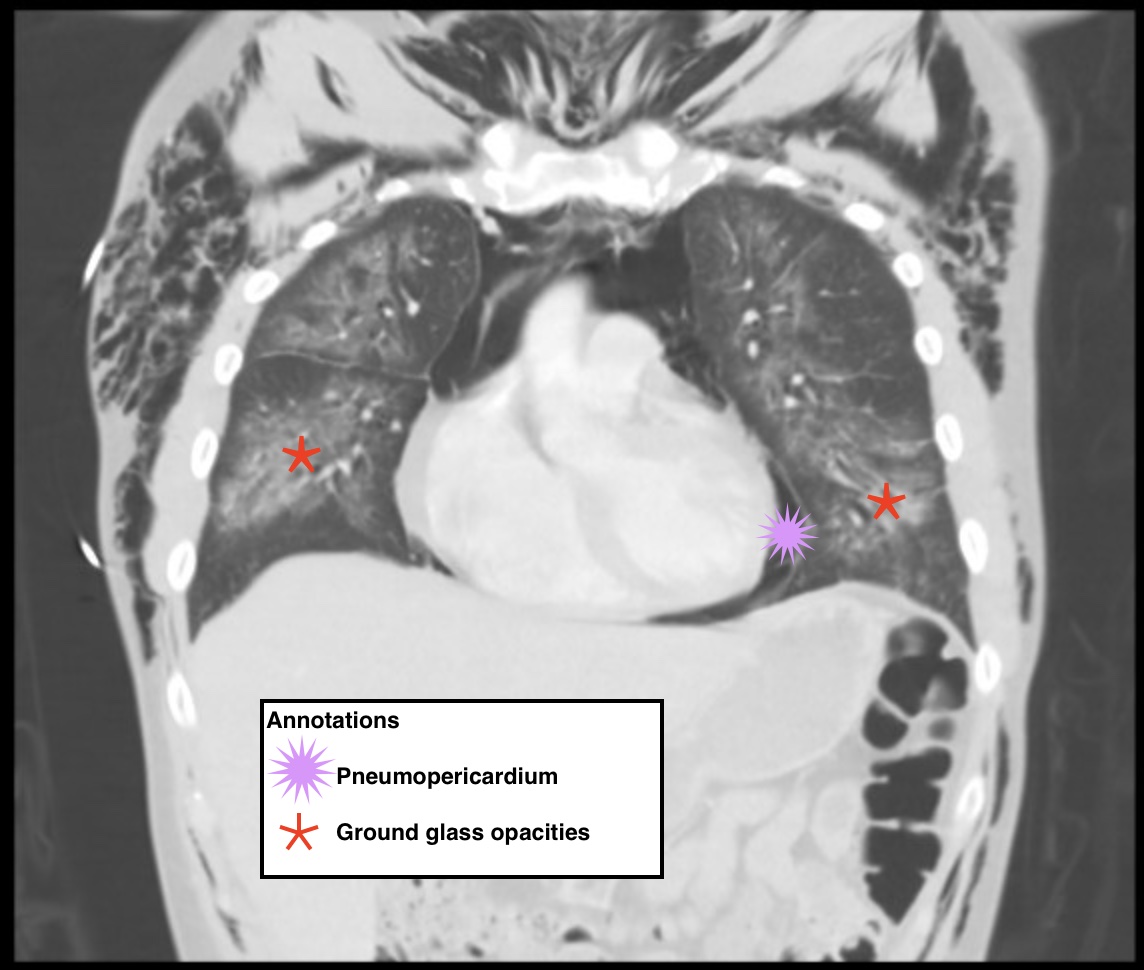

Supplement: Supplementary file 4 [file jetem-8-1-v22-supp3.jpg]

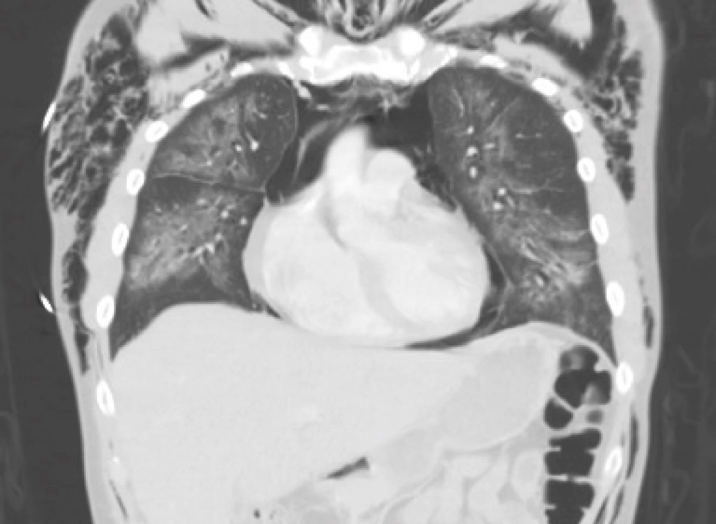

Supplement: Supplementary file 5 [file jetem-8-1-v22-supp4.jpg]
